# Chandipura Virus Forms Cytoplasmic Inclusion Bodies through Phase Separation and Proviral Association of Cellular Protein Kinase R and Stress Granule Protein TIA-1

**DOI:** 10.3390/v16071027

**Published:** 2024-06-26

**Authors:** Sharmistha Sarkar, Surajit Ganguly, Nirmal K. Ganguly, Debi P. Sarkar, Nishi Raj Sharma

**Affiliations:** 1 Department of Molecular Medicine, School of Interdisciplinary Studies, Jamia Hamdard University, Hamdard Nagar, New Delhi 110062, India; sharmisthasarkar_sch@jamiahamdard.ac.in (S.S.); surajit.ganguly@jamiahamdard.ac.in (S.G.); 2 Department of Education and Research, AERF, Artemis Hospitals, Gurugram 122001, India; nk.ganguly@artemishospitals.com; 3 Department of Biochemistry, University of Delhi South Campus, New Delhi 110021, India

**Keywords:** Chandipura virus, stress granules, phase separation, inclusion bodies, protein kinase R

## Abstract

Negative-strand RNA viruses form cytoplasmic inclusion bodies (IBs) representing virus replication foci through phase separation or biomolecular condensation of viral and cellular proteins, as a hallmark of their infection. Alternatively, mammalian cells form stalled mRNA containing antiviral stress granules (SGs), as a consequence of phosphorylation of eukaryotic initiation factor 2α (eIF2α) through condensation of several RNA-binding proteins including TIA-1. Whether and how Chandipura virus (CHPV), an emerging human pathogen causing influenza-like illness, coma and death, forms IBs and evades antiviral SGs remain unknown. By confocal imaging on CHPV-infected Vero-E6 cells, we found that CHPV infection does not induce formation of distinct canonical SGs. Instead, CHPV proteins condense and co-localize together with SG proteins to form heterogeneous IBs, which ensued independent of the activation of eIF2α and eIF2α kinase, protein kinase R (PKR). Interestingly, siRNA-mediated depletion of PKR or TIA-1 significantly decreased viral transcription and virion production. Moreover, CHPV infection also caused condensation and recruitment of PKR to IBs. Compared to SGs, IBs exhibited significant rapidity in disassembly dynamics. Altogether, our study demonstrating that CHPV replication co-optimizes with SG proteins and revealing an unprecedented proviral role of TIA-1/PKR may have implications in understanding the mechanisms regulating CHPV-IB formation and designing antiviral therapeutics. **Importance:** CHPV is an emerging tropical pathogen reported to cause acute influenza-like illness and encephalitis in children with a very high mortality rate of ~70%. Lack of vaccines and an effective therapy against CHPV makes it a potent pathogen for causing an epidemic in tropical parts of globe. Given these forewarnings, it is of paramount importance that CHPV biology must be understood comprehensively. Targeting of host factors offers several advantages over targeting the viral components due to the generally higher mutation rate in the viral genome. In this study, we aimed at understanding the role of SGs forming cellular RNA-binding proteins in CHPV replication. Our study helps understand participation of cellular factors in CHPV replication and could help develop effective therapeutics against the virus.

## 1. Introduction

Being an emergent tropical pathogen causing acute fever and encephalitis among children, Chandipura virus (CHPV) poses a huge threat to human health. First isolated in 1965 in Maharashtra State, India, from patients with febrile illness, CHPV, a member of the *Vesiculovirus* genus of the family *Rhabdoviridae* [1], is associated with severe human pathology which progresses rapidly from an influenza-like illness to coma and death [2]. CHPV came into the limelight in 2003, when the southern part of India experienced an outbreak of high mortality rate in which ~350 children developed acute encephalitis and ~200 died [2]. The proposed carrier of the virus is the female *phlebotomine* sandfly [2]. CHPV has a 150–165 nm long and 50–65 nm wide bullet-shaped morphology, as determined by transmission electron microscopy [3,4]. While a few CHPV outbreaks featuring a short incubation period and high mortality rate occurred in the past, their increased frequency in recent years raises serious concerns about CHPV and necessitates preparedness against this deadly virus [2]. CHPV genomic RNA is single-stranded and negative sense (11,119 nucleotides, nts) and contains a 49 nt leader gene (l) and five transcriptional units coding for viral polypeptides arranged in the order 3′ l-N-P-M-G-L-t 5′, separated by spacer regions and followed by a short non-transcribed 46 nt trailer sequence (t) [5]. The complete genome sequence of CHPV was determined recently and comparative analysis of its deduced protein sequences showed CHPV to be phylogenetically distinct from its prototype *Vesiculovirus*, vesicular stomatitis virus (VSV), but closely related to Isfahan virus (ISFV) [6].

Viruses are obligate parasites which hijack host cellular machinery for their multiplication and subsequent transmission. However, the embedded vital intracellular machineries are not easily accessible to viruses. To ensure their survival inside the host cells, viruses essentially need to counter the multiple layers of intracellular resistance to replicate and establish their dominance for their propagation [7]. One such layer in antiviral defense in mammalian somatic cells involves the formation of two types of RNA granules, processing bodies (P-bodies, PBs) and stress granules (SGs) [7,8,9]. Although sharing a few components, both these membraneless granules are physically and mechanistically distinct compartments with many unique biomarkers [10]. PBs are ubiquitously formed during normal conditions of cell growth and contain enzymes for RNA de-capping and degradation [8,11] and have been shown to store and degrade siRNA- or miRNA-guided mRNA [12,13]. SGs on the other hand are produced to store mRNA during cell stress conditions, lack de-capping/de-adenylating machinery and play a role in global translational arrest [8]. The function of SGs is thus to serve as a central and dynamic storehouse to protect stored mRNA species and exchange them with polysomes or PBs for further translation or degradation, respectively [11,14]. The formation of SGs is, in general, a defense mechanism against stress to sequester mRNAs and arrest translation in order to save metabolic energy during stress conditions. Besides containing several RNA-binding proteins [TIA-1, Ras-GAP (RasGTPase activating protein) SH3 domain-binding protein (G3BP1), Argonaute 2 (Ago2)], and mRNAs, SGs also contain 40S ribosomal subunits and also many translation initiation factors including eIF3, eIF4G, eIF4E and Poly(A)-binding protein PABP1 [11,15,16]. The exact mechanism of SG formation is, however, not very clear but involves reversible condensation of mRNA and RNA-binding proteins that stabilize or destabilize messenger RNA (mRNA).

In response to environmental stress, mammalian cells regulate translation initiation as an efficient mechanism to conserve metabolic energy and nutrients which are abundantly consumed during protein synthesis. One of the central mechanisms in this regulation to arrest translational initiation occurs by increasing the levels of phosphorylated eIF2α (p-eIF2α), which in turn leads to polysome disassembly triggering formation of SGs [17]. Among various kinases that phosphorylate eIF2α, host cell protein kinase R (PKR) acts as a sensor for foreign or viral double-stranded RNA (dsRNA) and after binding is activated to induce eIF2α phosphorylation leading to biomolecular condensation of RNA and proteins for SG formation [10,18]. This, in turn, shuts down host cell translation and triggers the host cell antiviral response [19,20]. However, viruses have evolved multiple strategies to bypass this response and even have been shown to utilize alternative ways of translation for their propagation [21]. Several viruses show the ability to suppress the formation of SGs [22,23,24,25,26]. For instance, poliovirus C3 protease and Semliki Forest virus nsP3 target G3BP, which regulates the initial phase of SG assembly [24,27,28,29]. Influenza virus NS1 inhibits the activity of dsRNA-activated PKR [22,23]. Interestingly, hepatitis C virus co-opts SGs and thus induces SG formation for its replication and production [30,31].

Several reports suggest that low-complexity sequence domains mediate the liquid–liquid phase separation in RNA-binding proteins which contribute to the formation of SGs or membraneless *biomolecular condensates* [32,33]. In the context of viruses, biomolecular condensation seems to provide viruses with numerous advantages that allow them to establish replication organelles, assemble viral particles and even evade innate immune responses [34]. It has been recently demonstrated that phase separation of the human adenovirus intrinsically disordered 52 kDa protein plays a critical role in the coordinated assembly of infectious progeny particles and for the organization of viral structural proteins into biomolecular condensates [35].

Recent studies suggested that, like SGs or PBs, viral IBs also have properties of biomolecular condensates [36]. We recently reported the varying degree of disorder in all five CHPV proteins, with the maximum level of intrinsic disorder propensity found in phosphoprotein (P) [5]. In pursuit of the identification of host factors involved in CHPV replication, we investigated three key aspects of CHPV infection. These included (1) understanding the propensity of CHPV proteins to undergo condensation and forming CHPV replication factories or inclusion bodies or IBs; (2) participation of cellular proteins in these factories and (3) CHPV evasion of the antiviral SG formation. Here, by immunofluorescence assay (IFA) on CHPV-infected cells for nucleocapsid (N) and large (L) protein detection, we found that CHPV proteins indeed show condensation and co-localize to form a heterogeneous population of puncta. However, CHPV infection did not induce distinct SGs but it promoted condensation of multiple SG markers (TIA-1, G3BP1, PABP1, Ago2 and eIF3η) which showed co-localization with large CHPV puncta, referred to as CHPV-IBs. Interestingly, recruitment of SG proteins to IBs ensued independent of PKR/eIF2α phosphorylation. Nevertheless, CHPV infection caused condensation and recruitment of PKR to IBs while siRNA-mediated depletion of PKR or TIA-1 significantly decreased virion production. The IBs exhibited cycloheximide sensitivity and, in comparison to SGs, significant haste in disassembly dynamics. Taken together, our study demonstrates TIA-1 and PKR as proviral factors in CHPV replication. This may have implications in understanding the mechanisms regulating CHPV-IB formation and designing antiviral therapeutics. Moreover, this study not only provides the first insight about the cytoplasmic viral IB formation during CHPV infection but also paves the way for a plethora of investigations to understand the mechanism of CHPV-IB formation during its replication.

## 2. Results

### 2.1. CHPV Infection Induces Condensation of Viral Nucleocapsid (N), Phosphoprotein (P) and Large (L) Proteins to Form Cytoplasmic IBs

We initiated this study by monitoring cytopathic changes in live CHPV-infected Vero E6 cells. Here, we observed a rapid change in the morphology of infected cells (CHPV MOI = 5), turning from elongated to round, as early as 4 h P.I. in ~5% of cells; by 7 h P.I., this could be detected in ~90% (Figure S1A,B). CHPV infection is known to induce apoptosis in a variety of mammalian host cells, although to varying degrees [37,38]. To understand the correlation between roundening and apoptosis in CHPV-infected Vero cells, we employed a commonly used tetrazolium dye based MTT assay to assess the cellular metabolic activity. Here, the time course data of escalating roundening of cells correlated with those of cell death and suggested roundening of cells precedes apoptosis in infected cells (Figure S1B).

**Figure 1:**
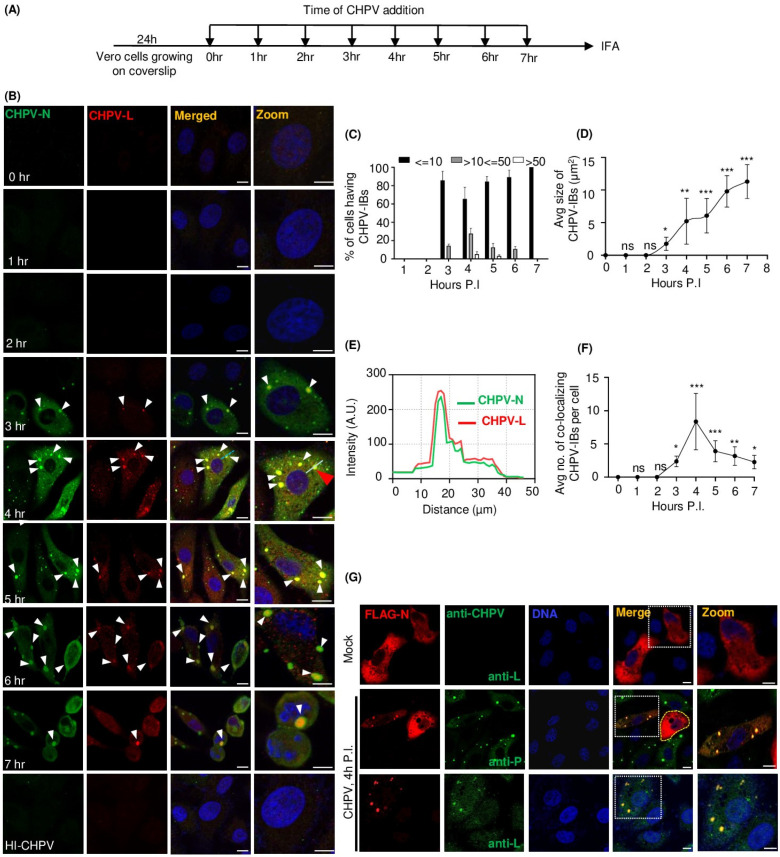
CHPV proteins condense and co-localize together to form cytoplasmic IBs in infected cells. (**A**) Outline of experimental design for the time course of CHPV infection in Vero cells grown on coverslips at the indicated time and infected cells were collected at the same time for further fixation and immunostaining. (**B**) Time course of CHPV infection. Vero cells were infected either with 5 MOI live CHPV for the indicated time or with heat-inactivated (56 °C/20 min) CHPV (HI-CHPV) for 7 h. Cells were fixed and co-immunostained for CHPV-N and CHPV-L using specific antibodies. The nuclei were counterstained with Hoechst dye. In the image, white arrows indicate CHPV puncta and red arrow indicate the punctate selected for line scan analysis. Scale bar = 10 µm. (**C**) Bar graph showing percentage of cells in (**B**) with less than or equal to ten (≤10), more than ten but less than or equal to fifty (>10≤50) or more than fifty (>50) CHPV-N puncta at each time point. The error bars represent SD from three independent experiments. ** *p* < 0.01 in Student’s *t*-test. (**D**) Graphical representation of the average size of CHPV-N puncta over the course of infection. CHPV-N puncta from at least 25 cells from each time point of infection were measured using ImageJ. The error bars represent SD from three independent experiments. *p* < 0.5 (*), *p* < 0.01 (**), *p* < 0.001 (***) in Student’s *t*-test. (**E**) The line scan analysis for the distribution of signal intensities for CHPV-N and CHPV-L over drawn line (shown by red arrowhead) in panel (**B**) (red arrow) using the ImageJ (version 1.53e) software. AU, arbitrary units. (**F**) Graph representing average no. co-localizing CHPV-IBs over the time course of CHPV infection calculated using ImageJ (n > 35 cells). The error bars represent SD from three independent experiments. *p* < 0.5 (*), *p* < 0.001 (***) in Student’s *t*-test. (**G**) CHPV-infection dependent condensation and co-localization of CHPV proteins. Vero cells were transfected with pFlagCMV6a-CHPV-N and 24 h later infected with 5 MOI CHPV. The cells were further co-immunostained for the detection of FLAG-tagged CHPV-N (using anti-FLAG) in combination with anti-P antibody or anti-L antibody for detection of phosphoprotein and L protein expressed during CHPV infection. Selected cells separated by dashed white borderlines from all three panels are shown as zoomed-in images. In the middle panel, CHPV-infected cells are separated by dashed white borderlines and uninfected cells outlined by yellow dashed lines. The nuclei were counterstained with Hoechst dye. Scale bar = 10 µm.

Next, to understand the propensity of CHPV proteins to undergo condensation during the course of infection, we performed IFA on CHPV-infected cells to detect expression and intracellular localization of two CHPV proteins (nucleocapsid “N” and large “L”) (Figure 1A). While N encapsidates the genome in a ratio of one N per nine ribonucleotides, protecting it from nucleases, L protein is an RNA-directed RNA polymerase that catalyzes the transcription of viral mRNAs, their capping and polyadenylation. Notably, N proteins are expressed abundantly and earlier compared to L protein, which is expressed late during CHPV infection. Here, by confocal microscopy, we observed expression of both proteins 3 h P.I., and expression of N protein was much more abundant than that of L protein, as expected (Figure 1B). Cytosolic heterogeneous expression of N protein was observed diffused as well as localized in puncta of variable sizes. However, we observed the maximum percentage of cells displaying the highest average number of CHPV-N puncta at 4 h P.I., with ~5–10% cells forming >50 puncta (Figure 1C).

The phenomenon of CHPV puncta formation in Vero cells appeared to be diverse, ranging from a maximum of 5–10 puncta/cell at 4 h P.I. (Figure 1B,F), which gradually decreased to as low as 1–2 puncta/cell at 7 h P.I., as small puncta coalesce to form large puncta by the end of 7 h, resulting in a steady increase in average size of puncta (Figure 1D). L protein, albeit expressed exclusively in the form of puncta, co-localized with large puncta of N (Figure 1B,D,E and Figure S1D), suggesting these to be the CHPV inclusion bodies, herein referred as “CHPV-IBs” or “IBs”.

In order to examine whether N protein can independently form puncta, we overexpressed a recombinant CHPV-N protein, fused to FLAG tag at its N-terminus, in Vero cells and confirmed its size and expression by an immunoblotting technique using anti-FLAG antibodies (Figure S1C). IFA on Vero cells ectopically expressing N protein, in the absence of CHPV infection, however, showed diffused distribution of recombinant N protein in the cytoplasm (Figure 1G, uppermost panel). CHPV infection in these cells, however, dramatically changed this distribution to localized in puncta which showed co-localization with both virally encoded CHPV-P and CHPV-L proteins (Figure 1G, middle and lower panels). Taken together, these results indicated that CHPV proteins (N, P and L) condense and co-localize to form cytoplasmic IBs during CHPV infection.

### 2.2. Recruitment of Multiple Stress Granule Proteins (SGPs) to CHPV-IBs

**Figure 2:**
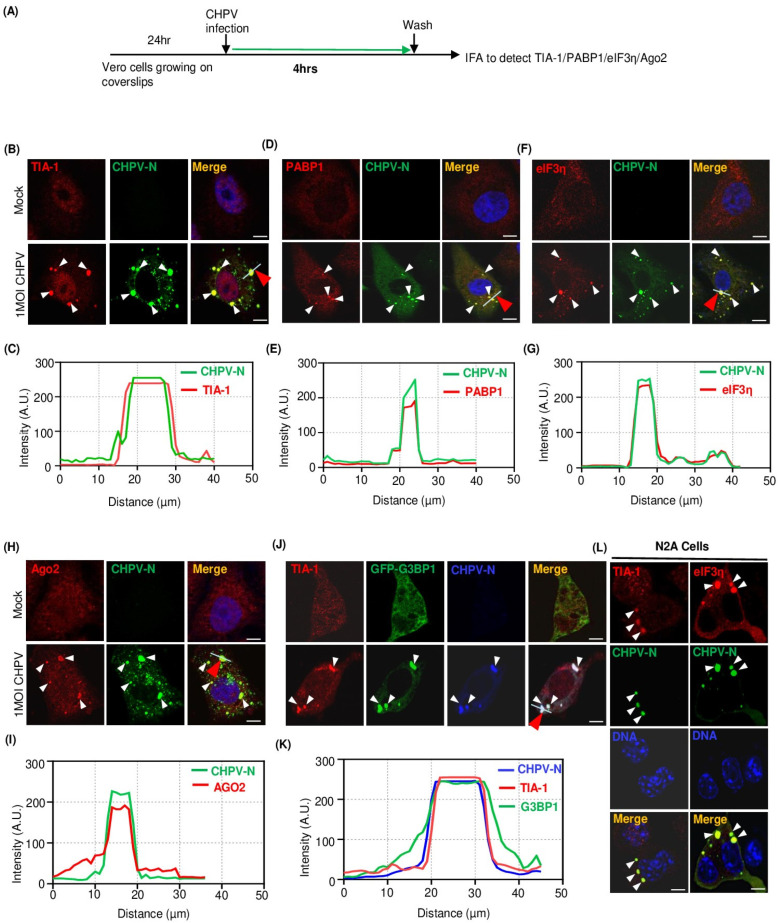
Multiple cellular SGPs associate with CHPV-IBs. (**A**) Outline of the experimental design for detection of SGPs in CHPV-infected cells. Vero cells grown on coverslips were infected with 1 MOI CHPV for 4 h and then processed for IFA to detect CHPV-N in combination with SGP using specific antibodies. (**B**–**I**) CHPV-IBs co-localize with endogenous SGPs (TIA-1, PABP1, eIF3η and Ago2). Vero cells were infected with 1 MOI CHPV for 4 h. Cells were then fixed with 4% PFA and immunostained for detection of endogenous markers. TIA-1 (red) in (**B**), PABP1 (red) in (**D**), eIF3η (red) in (**F**) and Ago2 (red) in (**H**) were detected along with CHPV-N (green) using specific antibodies. Graphical representations in (**C**,**E**,**G**,**I**) show the distribution of signal intensities across the line drawn across the CHPV-IB indicated with red arrow for each SGP and CHPV-N from panels (**B**,**D**,**F**,**H**) using the Image J software. AU, arbitrary units. The nuclei were counterstained with Hoechst stain. Scale bar = 10 µm. (**J**,**K**) Ectopic expression and co-localization of GFP-G3BP1 with CHPV-IBs. Vero cells transfected with GFP-G3BP1 or GFP alone (Figure S2D) expressing vector for 24 h were infected with 5 MOI CHPV for 4 h. The cells were stained for TIA-1 (red) and CHPV-N (blue) using their respective antibodies. Graphical representation in (**K**) shows the distribution of signal intensities of the line drawn across the CHPV-IB indicated with red arrow for GFP-G3BP1, TIA-1 and CHPV-N over selected cell from panel J) using the Image J software. AU, arbitrary units. Scale bar = 10 µm. (**L**) CHPV-IBs co-localize with endogenous SGPs in N2A cells. N2A cells were infected with 5 MOI CHPV for 4 h. The cells were then fixed with 4% PFA and immunostained for TIA-1 (red), eIF3η (red) and CHPV-N (green) using their respective antibodies. The nuclei were counterstained with Hoechst stain. Scale bar = 10 µm.

Virus infection, in general, causes stress on multiple biosynthetic pathways in host cells [39]. In response, mammalian somatic cells undergo global translational arrest and, in consequence, produce SGs to store stalled mRNA and prevent viral replication [8,40]. To understand whether CHPV infection induces SGs and their interplay with CHPV-IBs, we performed IFA on infected cells for several SGPs, as shown in the Figure 2A. Among these, TIA-1, an RNA-binding protein, recognizes the translationally arrested mRNPs and nucleates the assembly of SGs through its prion-like aggregation property [41]. As expected, in absence of CHPV infection, we observed predominant nuclear staining of TIA-1 but no visible distinct SGs (Figure 2B, upper panel). In contrast, CHPV-infected cells displayed TIA-1 forming 5–10 cytoplasmic puncta (Figure 1B lower panel). Since all TIA-1+ puncta also showed co-localization with IBs represented by CHPV-N immunostaining (Figure 2B,C), it was suggested that while CHPV infection does not induce formation of distinct canonical TIA + SGs but somehow induces TIA-1 localization to CHPV-IBs (Figure 2B,C). To address whether more SGPs are recruited to IBs, next we performed IFA to determine IBs’ association with three endogenous markers (PABP, Ago2, eIF3η) and one ectopically expressed (GFP-tagged G3BP1) marker of SGs. Here, Ago2 or Argonaute 2 is a core component of the RNA-induced silencing complex (RISC) to play its key role of catalytic engine that drives mRNA cleavage in RNA interference [42]. However, cellular stress leads to rearranged and increased association of Ago2 with the coding and 3′ UTRs of mRNAs and its recruitment to SGs [43,44]. RasGTPase-activating protein (GAP)-binding protein 1 (G3BP1) is a multi-functional RNA-binding protein, best known for its role in triggering the assembly and dynamics of SGs [10,45]. Likewise, eIF3η, a subunit of the eIF3 complex to control the assembly of the 40S ribosomal subunit and stabilizing eIF2-GTP-Met-tRNAiMet complex association, is also an authentic marker of SGs [46]. Interestingly, immunostaining of all the RNA-binding proteins (PABP1, eIF3η and Ago2), in contrast to their cytoplasmic diffused distribution in uninfected cells, exhibited puncta and their co-localization with CHPV-IBs (Figure 2D–I). Nevertheless, ectopic expression of GFP-tagged G3BP1 was observed in uninfected and CHPV-infected cells. When compared to its expected diffused distribution in uninfected cells, we found that G3BP1 also undergoes condensation and forms puncta in infected cells (Figure 2J, compare upper and lower panels). CHPV-N co-localization and TIA-1+ staining of these G3BP-1 puncta confirmed them as CHPV-IBs (Figure 2J,K). In contrast to GFP-G3BP-1, GFP alone, which served as a control protein, did not form visible puncta and also did not co-localize with IBs positive for TIA-1 and CHPV-N (Figure S2A,B).

Apart from SGs, mammalian cells also form lipid droplets, or LDs, as storage organelles at the center of lipid and energy homeostasis. They have a unique architecture consisting of a hydrophobic core of neutral lipids, which is enclosed by a phospholipid monolayer that is decorated by a specific set of proteins. LDs have been shown to be highly dynamic, ubiquitous organelles, which are found in virtually all types of cells from prokaryotes to eukaryotes. They consist mainly of triglycerides and sterol esters but also harbor other lipid species such as diacylglycerols, retinyl esters and ceramides [47,48], and can also activate the synthesis of bioactive lipid mediators [49]. LD biogenesis has been rapidly detected after infection with a number of different viral and non-viral pathogens [50]. Next, to understand any physical association between IBs and lipid droplets (LDs), we performed IFA using a BODIPY lipid droplet dye. This lipophilic dye can pass through the cell membrane and distribute within the cell, allowing it to specifically label cellular lipid droplets. Though we did not find co-localization of CHPV-IBs with LDs, there was a drastic drop in the number of LDs, suggesting that this effect could be associated with increased toxicity in the infected cells. Taken together, it appeared that IBs do not associate with LDs (Figure S2C,D).

CHPV exhibits neurological manifestation in young children and can induce neurological symptoms in suckling mice, thus indicating its neurotropic characteristics [51]. This led us to confirm CHPV neurospecificity for IB formation and their association with SG proteins in Neuro 2A (N2A) cells. Interestingly, similar to Vero cells, N2A cells displayed formation of ~3–5 CHPV-N IBs/cell which showed co-localization with condensed puncta of TIA-1 and eIF3η in cytoplasm (Figure 2L). In the absence of CHPV-N puncta in uninfected N2A cells, TIA-1 and eIF3η did not undergo visible condensation (Figure S2E). Notably, similar to GFP alone, GAPDH which also served as an endogenous control protein did not form visible puncta and also did not co-localize with IBs positive for CHPV-N (Figure S2F,G). Collectively, these results indicate that the ability of CHPV infection to trigger condensation and recruitment of several cellular SGPs to form IBs is cell-type independent. These results, however, do not distinguish IBs from canonical SGs.

### 2.3. CHPV-IBs and Canonical SGs Are Distinct in Terms of Disassembly Dynamics

**Figure 3:**
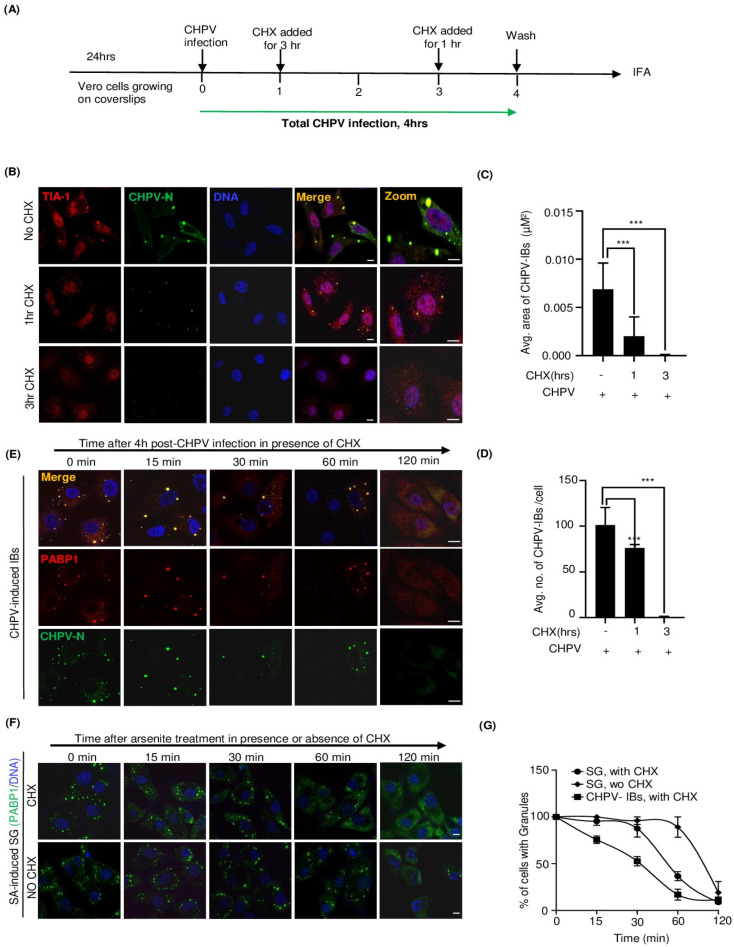
Cycloheximide (CHX) treatment reduces the size and number of CHPV-IBs. (**A**) The schematic shows outline of experimental design. Vero cells grown on coverslips were infected with CHPV 5 MOI for 4 h. CHX was added to cells at the indicated times for 3 h and 1 h treatment, respectively. (**B**–**D**) CHPV-IBs are sensitive to CHX treatment. Vero cells were infected with CHPV (5 MOI) either in the absence or presence of CHX (50 μg/mL) for 1 h and 3 h as shown in (**A**). After completion of 4 h of infection, cells were fixed with 4% PFA and immunostained for TIA-1 (red) and CHPV-N (green). The nuclei were counterstained with Hoechst dye. Scale bar = 10 µm. (**C**,**D**) Graphical representation of the number (**C**) and size (**D**) of CHPV-IBs with CHX treatment. *p* < 0.001 (***) in Student’s *t*-test. (**E**,**F**) Comparison of kinetics of CHPV-IB and SG disassembly upon CHX treatment. Vero cells were either infected with 5 MOI CHPV for 4 h (**E**) to induce IBs or treated with 1 mM SA for 40 min to induce SG (**F**). In both (**E**) and (**F**), cells were either left untreated or subsequently treated with CHX (50 μg/mL) for 15 min, 30 min, 1 h and 2 h to compare the dynamics of SG and IB disassembly. The cells were then fixed and co-immunostained for CHPV-N and PABP1. The nuclei were counterstained with Hoechst dye. Scale bar = 10 µm. (**G**) Graph showing comparison between the CHPV-IBs and canonical SGs with respect to their kinetics of disassembly in the presence of CHX. Untreated SGs without CHX served as a control.

Cycloheximide (CHX) is a translational inhibitor that stalls translation elongation without allowing the disassembly of the polysomes and thus prevents the formation and/or maintenance of SGs [52,53] Since viral replication exclusively relies on cellular translational machinery, we anticipated CHX to be a potential inhibitor of IBs and thought to use this ability to understand and compare the dynamics of assembly/disassembly of IBs with that of canonical sodium arsenite (SA)-induced SGs. In agreement with previous observations [14,54], SG induction by SA was inhibited in the presence of CHX (Figure S3A–C). Likewise, the size and number of IBs were also markedly reduced by incubation of CHPV-infected cells with CHX for 1 or 3 h (Figure 3A,B). Notably, the size of IBs dropped before their number, indicating the loss of dynamic equilibrium with the cytosolic protein components (Figure 3C,D). Altogether, these data suggested a close similarity between SGs and IBs in terms of assembly and, therefore, prompted us to compare the kinetics of their disassembly. To address that, Vero cells were either treated with SA for 40 min to induce SGs or infected with CHPV for 4 h to form IBs (schematics in Figure 3E,F). The medium of the treated or infected cells were replaced with a fresh medium to allow SGs and IBs to dissemble in the presence or absence of CHX and monitored by CHPV-N and PABP1 immunostaining followed by confocal microscopy. Here, in the absence of CHX, SGs disappeared at a very slow rate (~100 min for 50% reduction) (Figure 3F,G). However, in the presence of CHX, the rate of their disassembly became two times faster (~50 min for 50% reduction) (Figure 3E–G). In comparison, CHPV-IBs, the disassembly of which could only be imposed in the presence of CHX, interestingly exhibited a relatively faster rate of disassembly than SGs (~30 min for 50% reduction) (Figure 3F,G). Altogether, this implied not only the IBs’ dependence on viral mRNA translation for their induction and maintenance but also their higher sensitivity to CHX during the disassembly process, thus differentiating them from the canonical SGs.

### 2.4. TIA-1 and PKR Play a Proviral Role in CHPV Replication

**Figure 4:**
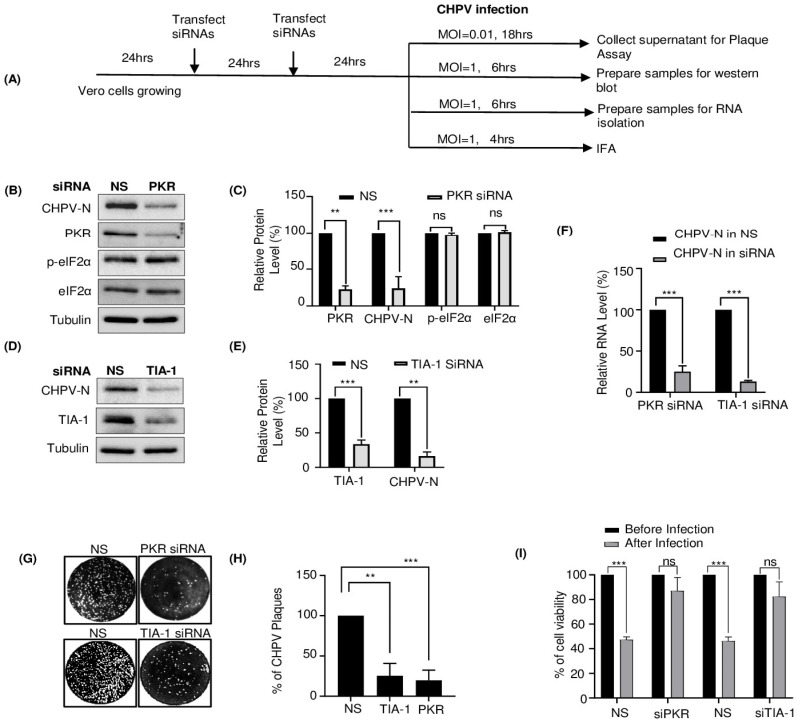
Silencing of TIA-1 or PKR expression decreases CHPV production. (**A**) Outline of experimental design for siRNA transfection following CHPV infection in Vero cells. After siRNA transfections (twice after an interval of 24 h), cells were equally divided into 4 sets. Each set was infected with the indicated MOI of CHPV for the indicated time for further detection of virions by plaque assay and quantification of viral RNA by qPCR and viral protein by Western blotting or IFA. (**B**) Western blot detection of PKR, CHPV-N, p-eIF2α, eIF2α and tubulin in CHPV-infected (1 MOI, 6 h) Vero cells after siRNA-mediated silencing of PKR expression. (**C**) Graphical representation of the relative amount of PKR, CHPV-N, p-eIF2α and eIF2α. The relative intensity of each protein band in PKR siRNA sample, after normalizing to tubulin, was calculated over that of the NS (Non-Specific) siRNA control. The error bar indicates mean ± SD (n = 3). *p* < 0.01 (**), *p* < 0.001 (***), ns = not-significant in Student’s *t*-test. (**D**) Western blot detection of TIA-1, CHPV-N and tubulin in CHPV-infected (1 MOI, 6 h) Vero cells after siRNA-mediated silencing of TIA-1 expression. (**E**) Graphical representation of the relative amount of TIA-1 and CHPV-N. The relative intensity of each protein band in TIA-1 siRNA sample, after normalizing to tubulin, was calculated over that of the NS siRNA control. The error bar indicates mean ± SD (n = 3). *p* < 0.01 (**), *p* < 0.001 (***), ns = not-significant in Student’s *t*-test. (**F**) Graphical representation of the relative amount of CHPV-N mRNA in CHPV-infected (1MOI, 6 h) Vero cells after siRNA-mediated silencing of PKR/TIA-1 as compared to that in NS siRNA control. The error bars indicate mean ± SD (n = 3). *p* < 0.01 (**), *p* < 0.001 (***), ns = not-significant in Student’s *t*-test. (**G**) Analysis of CHPV virion production after siRNA knockdown of PKR or TIA-1. As shown in (**A**), siRNA-transfected Vero cells were infected with CHPV (0.01 MOI) and allowed to replicate CHPV for 18 h. Cell culture supernatants obtained from the infected cells were used for plaque assay as described in Material and Methods. (**H**) Graphical representation of the quantification of plaques obtained in (**F**). Cells were fixed and stained with crystal violet at 18 h P.I., as described in Material and Methods. The error bar indicates mean ± SD (n = 3). *p* < 0.01 (**), *p* < 0.001 (***), ns = not-significant in Student’s *t*-test. (**I**) Graphical representation of the quantification of cell viability before/after CHPV infection (1 MOI, 6 h) in Vero cells after siRNA-mediated silencing of PKR/TIA-1 as compared to that in NS siRNA control. The error bars indicate mean ± SD (n = 3). *p* < 0.01 (**), *p* < 0.001 (***), ns = not-significant in Student’s *t*-test.

So far, we have found that CHPV infection induces condensation of TIA-1 and other SG proteins and association with IBs. To understand whether this association of TIA-1 is proviral or antiviral, we examined CHPV virion production in Vero E6 cells with or without siRNA-mediated TIA-1 knockdown. Although production of interferons is defective in Vero cells [55] which allows efficient viral replication, among its several effector molecules, double-stranded RNA (dsRNA)-dependent protein kinase (PKR), an enzyme with multiple effects in cells, is capable of playing a critical role in the antiviral defense mechanism of the host with important biological functions, including translational regulation [56]. Thus, in parallel, we also performed PKR knockdown to validate the hypothesis of the antiviral role of PKR in CHPV infection. By using TIA-1- or PKR-specific siRNAs, the dose of siRNA for a significant knockdown was optimized to achieve almost ~70% and ~85% of TIA-1 and PKR expression, respectively (Figure S4A–D). Next, we divided siRNA-transfected Vero cells into four groups to assess the effect of TIA-1/PKR depletion on CHPV gene expression and its replication as shown in Figure 4A. Cells were infected with 1 MOI for 4 or 6 h, as shown in Figure 4A, for cellular visualization of CHPV-N or its detection at the mRNA or protein level. Alternatively, cells were infected with low-MOI (0.01) CHPV for 18 h, followed by collecting supernatant for virion quantification using plaque assay. Here, we found that efficient knockdown of TIA-1 or PKR expression in Vero cells resulted in significantly decreased production of not only CHPV-N protein (Figure 4B–E) as detected by Western blotting but also of CHPV-N mRNA (Figure 4F) quantified by real-time PCR. It should be noted that silencing PKR expression did not change total levels of either eIF2α or its phosphorylated form, suggesting this effect to be specific to CHPV-N protein (Figure 4B,C). Moreover, IFA results corroborated with those of Western blotting showing that siRNA knockdown of PKR or TIA-1 expression in Vero cells led to a ~70–80% decrease in CHPV-N expression in the cells with the normal level of PKR or TIA-1 expression (Figure 4D,E and Figure S4E,F). Nevertheless, silencing of TIA-1 or PKR expression also led to a concomitant reduction of infectious virions in Vero culture supernatants (Figure 4G,H). Furthermore, to test whether the antagonistic effect of PKR or TIA-1 depletion on CHPV growth resulted in increased cell viability, we performed phase contrast microscopy and MTT assay to check cell viability. Here, we found decreased roundening and increased cell viability of CHPV-infected cells after TIA-1 or PKR knockdown (Figure 4I). Altogether, these data suggested TIA-1 and PKR play a proviral role in CHPV replication.

### 2.5. PKR Undergoes Condensation and Associates with CHPV-IBs

**Figure 5:**
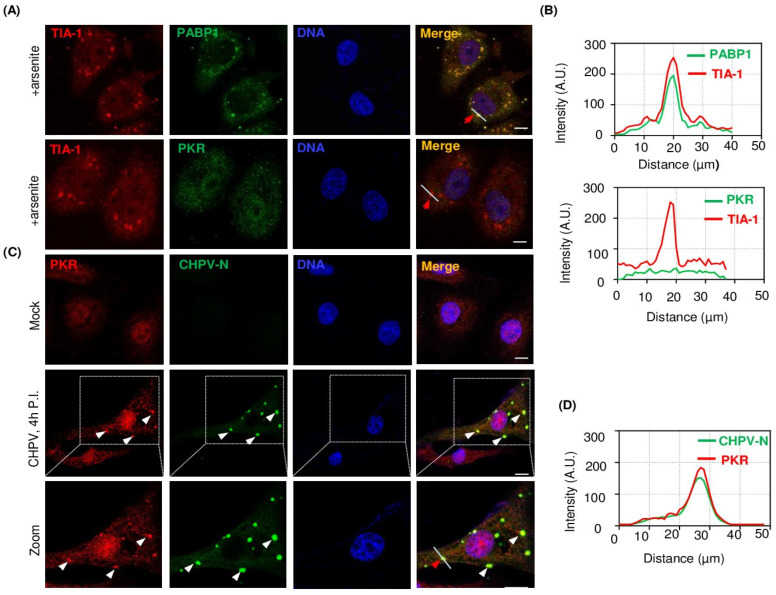
PKR associates with CHPV-IBs but not with SGs. (**A**) Vero cells were treated with 1 mM SA for 40 min to induce SGs. Later, cells were fixed with 4% PFA and processed for IFA to detect TIA-1/PABP1 or TIA-1/PKR for SGs. White arrows indicate CHPV-IBs. The nuclei were counterstained with Hoechst dye. Scale bar = 10 µm. (**B**) Graphical representations of the intensity plot across the line drawn in the images (**A**: upper and lower panels of SGs) for individual measurements in arbitrary units (AU) shown after normalization to maximum intensities using ImageJ software. (**C**) Vero cells were infected by CHPV 1 MOI for 4 h to induce IBs. Cells were fixed with 4% PFA and then processed for IFA to detect CHPV-N/PKR for CHPV-IBs. The nuclei were counterstained with Hoechst dye. Scale bar = 10 µm. (**D**) Graphical representations of the intensity plot across the line drawn across the CHPV-IB indicated with red arrow in the images ((**C**): lower panel of CHPV-IBs) for individual measurements in arbitrary units (AU) shown after normalization to maximum intensities using ImageJ software.

TIA-1 co-localization with CHPV-IBs made us speculate about a unique proviral role of PKR in CHPV replication and its participation in the formation of CHPV-IBs. To work on our hypothesis, we performed IFA on CHPV-infected Vero cells. In addition, to address whether this is a unique association of PKR with CHPV-IBs, we also sought to compare between IBs and canonical SGs. In this pursuit, we also treated one set of cells with 1 mM SA for 40 min to induce SGs. Both IBs and SGs were co-immunostained with anti-PKR antibodies. Additionally, SGs were also co-immunostained with anti-PABP1 antibodies together with anti-TIA-1 to compare and confirm the architecture of SGs. Here, in normal Vero cells, while PABP1 exhibited exclusively cytoplasmic immunostaining, both TIA-1 and PKR showed nucleocytoplasmic distribution (Figure S5A). SA-induced SGs stained positive for TIA-1 and PABP1 but stained negative for PKR (Figure 5A). The graphical representation of the measured signal intensity along a line across the SGs showed co-localization of TIA-1 and PABP1 but not of PKR (Figure 5B). On the other hand, CHPV-IBs, represented by CHPV-N immunostaining, also stained positive and displayed co-localization with PKR which, compared to uninfected cells, showed condensation in infected cells (Figure 5C,D). Taken together, the data suggested that similar to TIA-1, PKR also associates with IBs to play a proviral role in CHPV replication.

### 2.6. CHPV-IBs Form Independent of PKR and eIF2α Phosphorylation

**Figure 6:**
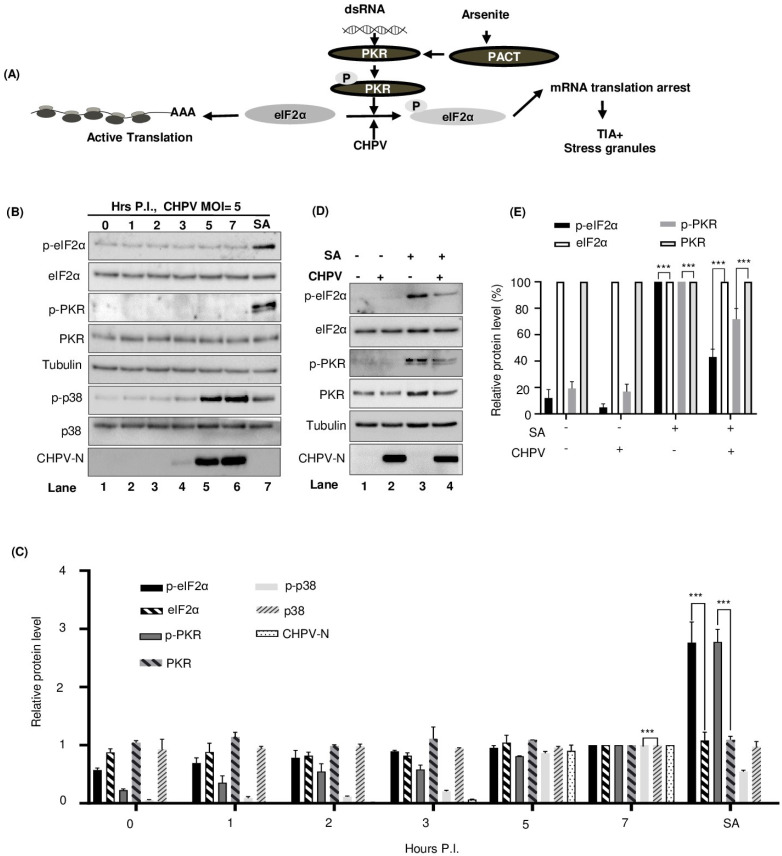
CHPV infection induces p38 phosphorylation but does not phosphorylate PKR/eIF2α. (**A**) Schematic diagram showing the cellular pathway p-PKR/p-eIF2α/SG governing SG formation. PKR direct activation by dsRNA or indirectly by SA through PACT is shown. (**B**) Kinetics of p38 activation with no change in p-PKR/p-eIF2α. Vero cells were infected with CHPV (5 MOI) for the indicated time. The phosphorylated forms of eIF2α (p-eIF2α, Ser51), PKR (p-PKR) and p38 (p-p38) were measured by Western blot analysis using phospho-specific antibodies. Total level of these kinases was determined by a pan-antibody. Tubulin served as a loading control. (**C**) Graphical representation of the relative amount of p-eIF2α, eIF2α, p-PKR, PKR, p-p38 and p38 protein in each sample after normalizing to tubulin was plotted over the time when the sample was collected (6B) with the protein level in lane 6 (7 h of CHPV infection) set as 1. The error bar indicates mean ± SD (n = 3). *p* < 0.001 (***) in Student’s *t*-test. (**D**,**E**) CHPV infection inhibits SA-induced eIF2α phosphorylation. Vero cells with or without CHPV infection were left untreated or treated with 0.5 mM SA for 30 min before cell lysate preparation for Western blotting with corresponding antibodies. CHPV-N was blotted as an indication for viral infection. (**E**) Relative amount of p-eIF2α, total eIF2α, p-PKR and total PKR in each sample after normalizing to tubulin was measured and plotted in bar graphs for comparison, with each protein level in lane 3 being set to 100%. The error bar indicates mean ± SD (n = 3). *p* < 0.001 (***) in Student’s *t*-test.

Phosphorylation of the α subunit of eIF2α stalls mRNA translation and promotes condensation and aggregation of TIA-1 to form the SGs for storing mRNA [57]. Of the four kinases which phosphorylate eIF2α (general control non-derepressible 2, GCN2; protein kinase R, PKR; PKR-like endoplasmic reticulum kinase, PERK and heme regulated inhibitory kinase, HRI), PKR can be phosphorylated by viral infection [20,58] and alternatively by SA [44,59]. While virus infection can generate dsRNA and activates PKR through binding of dsRNA to the dsRNA-binding domain (RBD) of PKR, SA activates PACT to bind and activate PKR [59,60] (Figure 6A). PKR possess at least 15 autophosphorylation sites, however, phosphorylation at Thr 446 and Thr 451 is critical for its activation and subsequent phosphorylation of eIF2α [61,62]. We hypothesized that, similar to SGs, condensation of SGPs and their recruitment to IBs during CHPV infection may be dependent on phosphorylation of eIF2α and also assumed PKR responsible for this function, possibly due to its association with IBs. Accordingly, we examined whether CHPV infection could affect PKR/eIF2α phosphorylation. To correlate kinetic production of CHPV proteins with both total eIF2α and phosphorylated eIF2α, we infected Vero cells with two doses of CHPV (1 MOI and 5 MOI) for the indicated time as shown in Figure 6B and Figure S6A. As mentioned in Figure 5B, we also treated uninfected Vero cells with 1 mM SA for 40 min before collecting cell lysates for Western blotting analysis to serve as a positive control for PKR and eIF2α phosphorylation. In this assay, while SA treatment induced phosphorylation of both PKR and eIF2α, CHPV infection did not induce such activity over that of uninfected cells (Figure 6B, lane 7 vs. lanes 1–6, and Figure 6C). It should be noted, however, that reprobing the same membrane with specific antibodies showed activation of p38 phosphorylation by CHPV infection, as reported earlier [63], with the total level of p38 protein remaining the same.

Next, we also wanted to understand the effect of CHPV infection on SA-induced PKR/eIF2α phosphorylation. For this, Vero cells uninfected or infected with CHPV were treated with SA and were analyzed by Western blotting for phosphorylation status of both eIF2α (p-eIF2α) and PKR (p-PKR). As expected, SA was found to induce eIF2α phosphorylation that was ~10 times greater than the basal level (Figure 6D, lane 3 vs. lane 1). In contrast, CHPV-infected cells exhibited remarkable inhibition (~60%) of eIF2α phosphorylation upon SA induction (Figure 6D, lane 4 vs. lane 3, Figure 6E), with no detectable changes in the total eIF2α protein signal. Moreover, a similar effect of CHPV infection on partial inhibition of PKR phosphorylation (~30%) was also observed without any effect on total PKR levels (Figure 6D, lane 4 vs. lane 3, Figure 6E). These data indicated that CHPV infection is partially inhibitory for PKR and eIF2α phosphorylation. Taken together, the data suggested that condensation and recruitment of SGPs to CHPV-IBs are independent of PKR/eIF2α phosphorylation.

## 3. Discussion

Despite its huge potential to cause a deadly infection, how CHPV utilizes or hijacks cellular factors to replicate inside cells remains largely unknown. Negative-strand RNA viruses, in general, induce the formation of cytoplasmic inclusion bodies (IBs) which involves phase separation of viral and cellular proteins. Numerous reports recently came out to suggest viral proteomes contain intrinsically disordered proteins (IDPs) and IDP regions (IDPRs), which are proteins or protein regions that lack unique (or ordered) three-dimensional structures [5,64,65]. Of late, a number of reports also suggested involvement of IDPs and IDPRs in cell signaling [66], assembly of cellular SGs [67,68] and condensation and aggregation of viral and cellular proteins to form IBs [69]. In this direction, we published a report emphasizing the varying degrees of disorder in all five CHPV proteins, with the maximum level of intrinsic disorder propensity found in phosphoprotein (P) [5]. To further investigate the propensity of CHPV proteins for phase separation and for exploration of its host factor usage, a Vero cell line, as reported earlier [38], was investigated as a suitable host system for CHPV replication. While the cytotoxicity assay on CHPV-infected cells helped to understand the timeframe and dynamics of CHPV gene expression (Figure S1), IFA on these cells allowed us to monitor condensation and punctate localization of viral proteins to form CHPV-IBs (Figure 1). Although sometimes identified with a different name, e.g., Negri bodies in rabies virus, IBs are consistent among members of *Rhabdoviridae* as well as among negative-strand RNA viruses [36,54,70]. For instance, a closely related member of the family, VSV, forms TIA-1+ SG-like structures that co-localize with viral replication proteins and RNA [54]. Virus infection, in general, inevitably induces metabolic stress and promotes SG production. Thus, in pursuit of identification of host factors, we performed this study to understand CHPV-IB formation and the regulation of SGs in this course. We found that CHPV infection did not induce formation of distinct SGs but showed association of its IBs with multiple RNA-binding proteins of SGs (TIA-1, PAPB1, Ago2, eIF3η and G3BP1) (Figure 2). We anticipate involvement of more SGPs in CHPV-IB formation and think it would be interesting to characterize IBs further to gain more insight about their composition with respect to cellular proteins. In this direction, whether assembly of IBs is triggered by scaffolding activity of a cellular or a viral component would be another important question to address in future investigation. CHPV phosphoprotein, a mostly intrinsically disordered protein at its N-terminus, as found in our previous study [5], might also be a potential factor to be investigated further for this role and in understanding the mechanism of CHPV-IB formation.

Our investigation subsequently focused on differentiation of IBs with canonical SGs. Although SGs are often assumed to be uniform entities formed under different stresses, their protein and mRNA compositions vary [71]. The formation of SGs is a dynamic and reversible assembly which disintegrates and cellular translation resumes when the cell starts to recover from stress. To understand the dynamic properties and to address whether already formed IBs can be strained enough to disintegrate by limiting the production of its constituents, we first tested CHX as an inhibitor of CHPV-IBs which has been already demonstrated earlier for SGs [71]. Here, we found CHX can effectively reduce the size and exhibit enforced disassembly of IBs by 3 h of its treatment in CHPV-infected cells (Figure 3). The inhibitory effect of CHX on IBs not only confirmed IBs to be virally induced but also allowed us to identify the time of their disassembly. When compared to SA-induced conventional SG, IBs were found to be different than SGs in terms of kinetics of their disassembly in the presence of CHX. Altogether, it indicated that IBs are formed through a different process than SGs and they might have a different composition and architecture and thus different kinetics of disassembly than conventional SGs.

Next, we aimed to understand whether TIA-1 is a provirally or antivirally associated with CHPV replication. TIA-1 is a key player in the formation of SGs which in turn trigger the antiviral cellular response and limit virus production [40]. However, TIA-1 also exhibits a proviral role in the replication of *flaviviruses* [72], so its antiviral effect is not universal for all viruses. Since PKR is widely accepted to play an antiviral role in infection by many viruses of several families [73], we postulated that PKR might be a host inhibitory protein to block CHPV production. To our surprise, independent silencing of TIA-1 or PKR in Vero cells showed similar effects of a significant decrease in CHPV virion production and suggested a proviral role of both TIA-1 and PKR (Figure 4). The proviral role of TIA-1 in CHPV replication bears a resemblance to its supportive role in *Flavivirus* genome RNA synthesis through interaction with viral components and inhibiting SG formation [25]. Our observation through IFA on CHPV-infected cells also revealed co-localization of PKR with CHPV-IBs but not with SGs (Figure 5). Here, it should be noted that PKR’s association with IBs makes them different than SGs in terms of their composition. In this context, CHPV resembles porcine reproductive and respiratory syndrome virus (PRRSV), where PKR has been demonstrated to play a proviral role in viral replication by modulating viral gene transcription [74]. It was also demonstrated later that PRRSV selectively inhibits PKR activation to prevent inflammatory response. Interestingly, this inhibition of PKR was shown to be dependent on the viral nsp1β protein to co-optimize G3BP to inhibit PKR activation in viral replication factories [75]. Further investigation is needed to understand the mechanism of proviral function of both TIA-1 and PKR.

While the process of IBs remains completely unknown, the canonical SG formation is initiated as a consequence of phosphorylation of the α subunit in elF2α at a specific serine (Ser 51) residue [76]. The phosphorylation of eIF2α acts as a trigger which causes a prion-like aggregation, phase separation and recruitment of TIA-1 to SGs [77]. Typically, eIF2α functions as a vital initiation factor in promoting the binding of tRNA^met^ to the 40S ribosome to promote mRNA translation in a GTP-dependent manner. Different types of stress (oxidative, heat or nutrient deprivation) can induce eIF2α phosphorylation by activation of four different eIF2α kinases (GCN2, PKR, PERK and HRI) [57].

**Figure 7:**
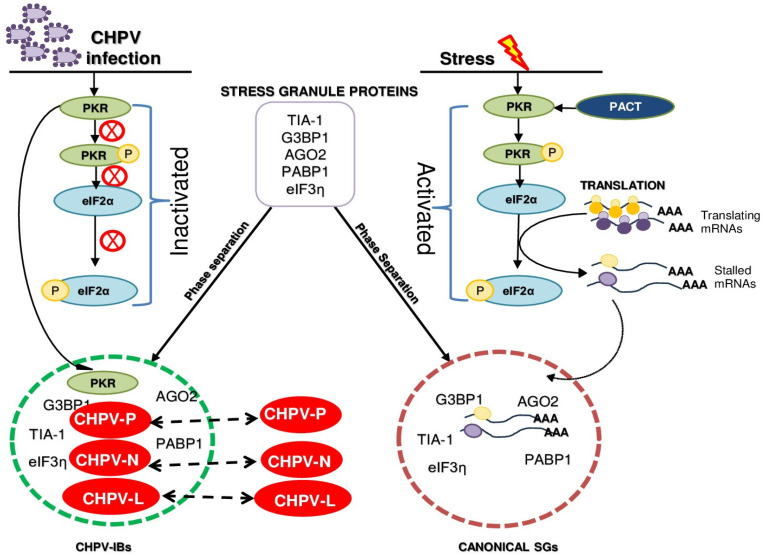
A schematic model showing distinctness in the process of SG and CHPV-IB formation. In this model, CHPV proteins (N, P and L) in the cytoplasm condense and co-localize together in association with other viral proteins, cellular SG proteins and PKR to form CHPV-IBs. In contrast to formation of SGs, IBs form independent of the activation of PKR/eIF2α phosphorylation.

To further compare the process of IB formation with SGs, our investigation focused on the activation of PKR/eIF2α for recruitment of TIA-1 and other RNA-binding proteins to CHPV-IBs, as happens during SG formation. Here, we found CHPV infection could neither activate PKR nor induce eIF2α phosphorylation, thus suggesting that the recruitment of TIA-1 and other RNA-binding proteins to CHPV-IBs occurs independent of activation of this pathway (Figure 6). Our observation on partial inhibition of PKR and eIF2α phosphorylation in SA-treated and CHPV-infected cells may be related to condensed PKR in IBs remaining inactive and inaccessible to PACT-mediated activation during SA treatment. Taking everything together, it can be concluded that CHPV-IBs and SGs are distinct in terms of their disassembly, composition and in the process of their formation. Our proposed model in Figure 7 shows the two distinct pathways where activation of the PKR pathway occurs in the formation of SGs but not in the CHPV-IBs. PKR in its inactive form, however, plays an unknown proviral function in the formation of IBs. Moreover, the presence of viral proteins (shown in red) in IBs and presumably viral RNA make their composition different from SGs. In addition to viral RNA, its regulated transportation and translation, it will also be interesting to know about the presence of cellular mRNA in IBs. Altogether, this study not only provides insight into CHPV replication and proposes several important questions to understand CHPV biology but also lays the foundation for designing antiviral therapy against CHPV.

## 4. Material and Methods

*Cell cultures and virus propagation*. Vero E6 cells (ATCC, Manassas, VA, USA) and N2A cells were cultivated in DMEM (cat no. 12430047, GIBCO/Thermofisher scientific, Waltham, MA, USA) supplemented with 10% heat-inactivated fetal bovine serum (cat no. 16140-071, GIBCO/Thermofisher scientific). Human-patient-derived CHPV (strain no. 1653514) was obtained from Dr. Dhrubajyoti Chattopadhyay’s lab (Calcutta University). The virus was propagated in the Vero cells, and viral titer (5 × 10^7^ pfu/mL) was measured using plaque assay.

*Antibodies and chemicals.* The antibodies used for this study and their respective working dilutions in Western blotting (WB) or immunofluorescence assay (IFA) are as follows. Rabbit polyclonal anti-CHPV-N (1:5000, WB and 1:250 IF), anti-CHPV-P (1:5000, WB and 1:250 IF) and mouse anti-CHPV-L (IF 1:250) antibodies were kindly provided by Dr. Dhrubajyoti Chattopadhayay. Mouse anti-TIA-1 (cat no. SC-166247), rabbit anti-p38 (cat no. SC-728 and goat anti-eIF3η (cat no. SC-16377) were obtained from Santa Cruz Biotechnology (Dallas, TX, USA). Rabbit anti-eIF2α (cat no. 9722S), rabbit anti-phospho-eIF2α (Ser 51) (cat no. 9721S) and rabbit anti-phospho-p38 (cat no. 9211S) were obtained from Cell Signaling Technology (Danvers, MA, USA). Mouse anti-AGO2 (cat no. 57113, Abcam, Cambridge, MA, USA), mouse monoclonal anti-eIF2AK2 (PKR) (cat no. H000005610-M01, Abnova, Taipei, China), rabbit anti-phospho-PKR (pThr454) (cat no. 527460, Millipore), mouse anti-PABP1 (cat no. MA1 34079/Invitrogen/Thermofisher scientific) and mouse anti-β-tubulin (cat no. T5201, Sigma Aldrich, St. Louis, MO, USA) were obtained from their respective companies. The secondary antibodies Alexa Fluor 568 (H+L) donkey anti-goat (cat no. A11057), Alexa Fluor 488 (H+L) goat anti-rabbit (cat no. A11008), Alexa Fluor 568 (H+L) goat anti-mouse (cat no. A11004) and Alexa Fluor 405 (H+L) goat anti-rabbit (cat no. A31556) were obtained from *Thermofisher scientific*.

*Induction of cellular stress.* Sodium arsenite (SA, cat no. 1062771000, Sigma Aldrich) solution (0.05 M) was used. To induce oxidative stress, the cells were cultivated in fresh culture medium containing 1 mM SA for 40 min [18].

*Immunofluorescence assay.* Adherent Vero E6 cells were grown directly on glass coverslips. Immunofluorescence staining was performed as described previously [18,44,78]. Briefly, the pretreated or infected cells were washed with PBS, fixed with 4% paraformaldehyde (PFA), permeabilized with 0.4% Triton X-100 and blocked with 2% bovine serum albumin (BSA, cat no. A9647, Sigma Aldrich) dissolved in Tris-buffered saline containing 0.05% Tween-20 (TTBS). Primary antibodies diluted in blocking buffer were incubated with slides overnight at 4 °C or 3 h at 37 °C in a humidified chamber. Alexa-Fluor-conjugated secondary antibodies (1:500, ThermoFisher Scientific) were diluted in blocking solution and incubated with slides at 37 °C in humidified chamber for 2 h. The slides were washed at least 4 times with TTBS and, before mounting the cells, nuclei were visualized by 5 min counterstaining with wash buffer containing Hoechst dye 33342 (1:10,000 dilution, cat no. B2261 Sigma-Aldrich).

*Staining of cellular lipid droplets.* A stock solution of 10mg/mL (38 mM) BODIPY™ 493/503 (4,4-Difluoro-1,3,5,7,8-Pentamethyl-4-Bora-3a,4a-Diaza-s-Indacene/cat no. D3922, ThermoFisher Scientific) was prepared in DMSO (1:100) to make a substock of 0.1 mg/mL (0.38 mM). This was further diluted in PBS (2.5 µL in 1 mL PBS) to make working solution of 1 µM to stain neutral lipids in cells.

*Expression vectors and construction of plasmids.* The following vectors were used to express recombinant proteins: FLAG-tagged CHPV-N (Chandipura virus nucleoprotein) expressed by a mammalian expression vector was generated by subcloning the CHPV-N gene from the PET-3a-CHPV-N plasmid (a gift from Dr. Dhrubajyoti Chattopadhayay) in pFLAG-CMV6a (Sigma). Primers F 5′-TTTATA AAGCTT ATGAGTTCTCAAGTATTC-3′ and R 5′-TTTATA GGATCCTCATGCAAAGAGTTTCCT-3′ containing the Hind III and Bam HI sites, respectively, were used to amplify the CHPV-N gene. The PCR product was subcloned in between sites Hind III and Bam HI in MCS of pFLAG-CMV6a plasmid with a CMV promoter. The recombinant plasmid was purified using a QIAgen mini prep kit (cat no. 27106, Qiagen, Hilden, Germany) as per the manufacturer’s protocol. The resulting plasmid, subsequently named as pFlagCMV6-N (pNRS1), was verified by restriction digestion and sequencing. The GFP-G3BP1 wild type construct was kindly provided by Dr. Jomon Joseph (NCCS, Pune, India), originally obtained from Dr Jamal Tazi (Institut de Génétique Moléculaire de Montpellier, France). A GFP-expressing plasmid (pEGFP-N1) was obtained from Clontech (cat no. 6085-1).

*Plasmid transfection.* Plasmid transfections were performed using Lipo2000 transfection reagent (cat no. 11688-030, Invitrogen by ThermoFisher Scientific) according to the manufacturer’s instructions. Unless indicated, for IFA and Western blot analysis, Vero E6 cells (3 × 10^5^) were plated a day prior to which 1 µg of plasmid DNA transfection in a 6-well plate was performed.

*siRNA transfection and measurement of CHPV virion production.* For siRNA-mediated silencing, ~1.5 × 10^5^ Vero E6 cells growing in a 12-well plate were transfected twice at an interval of 24 h with siRNAs targeting human PKR/EIF2AK2 (Assay ID 142330-Thermofisher scientific) or human TIA-1 (Assay ID 139893-Thermofisher scientific) or GFP-targeting siRNA as a negative control (cat no. P-002048-01-20, Dharmacon, Lafayette, CO, USA) using Lipofectamine 3000 transfection reagent (cat no. L3000-008-Invitrogen/Thermofisher scientific). Total cell extract was collected 24 h after the second siRNA transfection to measure the knockdown efficiency by Western blotting.

Alternatively, for CHPV virion production and titration assays, Vero cells in a 12-well plate were transfected with siRNAs targeting human PKR or TIA-1 and after 20 h of a second round of siRNA transfections as described above, they were infected with 0.01 MOI CHPV in 1 mL of DMEM. Culture supernatants were harvested 18 h after infection, cleared by centrifugation at 380× *g* (2000 rpm) for 10 min and aliquoted for further measurement of viral titer by plaque assay. In parallel, in three plates of siRNA-transfected cells, 1 MOI CHPV was incubated in 1 mL of complete medium. In one of the plates, Vero cell extract was prepared after 6 h of infection as mentioned below for Western blotting to detect viral CHPV-N or cellular PKR or TIA-1, p-eIF2α, total eIF2α and tubulin using specific antibodies. In another plate, after 6 h of infection, Vero cells were harvested for RNA extraction to measure the amount of viral RNA. In yet another parallel plate of siRNA-transfected cells, 1 MOI CHPV was incubated in 1 mL of complete medium. After 4 h of infection, Vero cells were fixed using 4% PFA and later immunostained for CHPV-N or cellular PKR or TIA-1.

*Plaque assay.* Vero cells (~1.5 × 10^5^ cells/well) were grown in 12-well plates and allowed to grow for 48 h to reach confluency of ~90–95%. Cells were washed twice with 1X PBS and added with cell-free supernatant containing CHPV virions diluted in serum-free medium and incubated for 2 h at 37 °C in a CO_2_ incubator. Again, cells were washed with PBS twice and overlaid with 2× DMEM mixed with an equal volume of 2% low-melting-point agarose. Plates were then incubated for 18–24 h at 37 °C in a CO_2_ incubator. To visualize plaques, cells were stained with crystal violet (0.5% *w*/*v* crystal violet in 25% methanol) for 2 h and the agarose overlay was discarded. Finally, wells were rinsed with water for visual counting of the plaques.

*Western blotting*. Unless indicated otherwise, protein samples for Western blots were prepared by direct lysis of the cells in 2X SDS sample buffer (0.02% bromophenol blue (*w*/*v*) in 4%SDS, 120 mM Tris HCl pH = 6.8 and 20% glycerol (*v*/*v*)) containing 5% 2-mercaptoethanol (Sigma-Aldrich). Samples were resolved on a 10% SDS-PAGE gel in 1 × Tris glycine SDS buffer. The signal was detected with chemiluminescent substrate (BIORAD, Hercules, CA, USA, cat no. 1705061).

Densitometric quantification of Western blots was performed using ImageJ (version 1.53e) software through the rectangular selection tool. Mean density of the selected area was calculated after selecting all the bands.

*RNA extraction, cDNA preparation and qRT-PCR.* Vero E6 cells growing in 12-well plates after siRNA transfection and/or infection with CHPV were lysed in 1 mL Trizol (cat no. 15596026, Invitrogen/Thermofisher scientific), 200 μL of chloroform was added and mixed vigorously for 15 s and the mixture was incubated at RT for 2–3 min. The mixture was centrifuged at 13,000 rpm for 15 min at 4 °C to separate out aqueous and phenol layers. The aqueous layer was separated carefully; an equal amount of isopropanol was added and incubated on ice for 30 min to precipitate the RNA. The precipitated sample was centrifuged at 16,200× *g* (13,000 rpm) for 15 min at 4 °C. The RNA pellet was washed using 70% ethanol and then resuspended in 15 μL of nuclease-free water. DNase treatment was given to the extracted RNA for 15 min at RT using a DNase-1 kit (cat no. 18068-015, Gibco/Thermofisher scientific) according to the manufacturer’s protocol. The DNA-free RNA was used for cDNA preparation using a Takara first strand cDNA synthesis kit (cat no. 6110A, Takara Bio Inc., Kusatsu shi, Japan) according to the manufacturer’s protocol. The RNA template of ~1 µg was incubated with oligo dT primers (50 µM) and dNTP mixture (10 µM) at 65 °C for 5 min and then 5 min on ice. Later, the template RNA/primer mixture was combined with 5X Prime script buffer, 20 U of RNase inhibitor and 200 U of Prime script reverse transcriptase and underwent PCR (30 °C for 10 min, 42 °C for 60 min and 72 °C for 15 min) to synthesize first-strand complementary DNA. The prepared cDNA (0.25 µL) was used for quantitative real-time PCR using 2× SYBR green master mix (cat no. 4344463, applied biosystems/Thermofisher scientific) to measure the amount of CHPV-N-specific mRNA in infected Vero cells using CHPV-N-specific primers (forward 5′-ACCTGGCTCCAAATCCAATAC-3′ and reverse 5′-GGTGGATCAGACGGAGAGATA-3′). β-actin (forward 5′-GACAGGATGCAGAAGGAGAT-3′ and reverse 5′-GCTTGCTGATCCACATCTGC-3′) was used as the housekeeping gene.

*Cycloheximide treatment*. To understand effect of CHX on SG formation, Vero cells were grown on coverslips in three 35 mm dishes. One of the dishes was pretreated with 100 µg/mL of CHX for 1 h; later, media were replaced with complete media containing 1 mM SA for 40 min in the presence of CHX. Another dish was treated only with 1 mM SA for 40 min. Yet another dish was not treated at all and served as a control. All 3 plates were fixed with 4% PFA and stained for TIA-1 and PABP1 using their respective antibodies.

To understand the effect of CHX on CHPV-IBs, Vero E6 cells were grown on coverslips in 6-well plates in two sets and allowed to reach confluency of 70–80%. Cells were infected with 2 MOI CHPV and after 3 h of infection virus-containing media were replaced with complete media containing 100 µg/mL of CHX. After 1 h of CHX treatment, cells were fixed with 4% PFA and used for IFA. Another set of cells were infected with 2 MOI CHPV and after 1 h of infection, virus-containing media were replaced with complete media containing 100 µg/mL of CHX. After 3 h of CHX treatment cells were fixed with 4% PFA and used for immunofluorescence staining.

To understand the dynamics of CHPV-IBs in contrast to SGs through inhibition of protein synthesis by cycloheximide (CHX), Vero E6 cells were grown on coverslips in 35 mm dishes and allowed to reach confluency of 70–80%. Cells were infected with 2 MOI CHPV, and after 4 h of virus infection cells were treated with 100 µg/mL of CHX for 15 min, 30 min, 1 h and 2 h, respectively. Then, they were fixed with 4% PFA and used for immunofluorescence staining. Another set of cells were first exposed to 1 mM of SA for 45 min to form canonical stress granules and then treated with 100 µg/mL of CHX for 15 min, 30 min, 1 h and 2 h, respectively. Then, cells were fixed with 4% PFA and used for immunofluorescence staining.

*MTT assay.* To understand the metabolic state of cells upon CHPV infection, an MTT assay was used. Vero cells were plated in 96-well plates and allowed to reach confluency of 70–80%. Cells were infected with 5 MOI CHPV at different time points; after completion of virus infection virus-containing media were replaced with media containing 5 µg/mL MTT reagent (thiazolyl blue tetrazolium bromide, cat no. 298-93-1, Gold biotechnology, St. Louis, MO, USA). It was incubated at 37 °C for 3–4 h, then MTT-containing media were removed and 100 µL of DMSO was added to each well to dissolve the formazan crystals formed. After 15 min, OD was measured at 594nm. The amount of formazan formed is directly correlated to live cells in the well.

*Confocal imaging and statistical analysis:* Fluorescence images were captured with Leica SP8 confocal microscope equipped with a 63X oil immersion objective lens. The images were processed in Las X free software (Leica Application Suite X version 3.7.6.25997-2022).

Data were represented as the mean of 3 biological replicates ± standard deviation from the mean. Student’s *t*-test was performed in GraphPad Prism 8 to evaluate the statistical significance. A *p*-value of <0.05 was considered statistically significant.

Line scan analysis of CHPV-IBs was performed in ImageJ to quantify intensities across a line dissecting a particular CHPV-IB. All three channels (red, green and blue) of image files were opened and then merged. In the merged image a line dissecting a particular CHPV-IB was drawn using the line tool, then the “RGB Profile Plot” plugin was selected to plot signal intensities across the line.

Quantification of CHPV-IBs was performed in ImageJ. To count and quantify CHPV-IBs the “Analyze Particle” plugin of ImageJ was used for which images were converted to 8-bit gray-scale, scale was set to 10 μm and threshold was set to eliminate the background and select only CHPV-IBs. Each cell was then marked using the “Free Hand Tool” and the Analyze Particle plugin was applied to analyze the number of CHPV-IBs per cell and the size of each CHPV-IB inside the cell. To calculate the Pearson’s correlation analysis of co-localizing IBs, TIFF images of red and green channels were opened in ImageJ individually and merged. The merged image was converted to RGB color format, and the color threshold was set to select only co-localizing IBs. Total co-localizing area was measured and the JACop plugin of ImageJ was used to obtain the Pearson’s correlation coefficient.

## Data Availability

All data is available upon reasonable request.

## Supplementary Materials

The following supporting information can be downloaded at: https://www.mdpi.com/article/10.3390/v16071027/s1, **Figure S1A:** Time course of CHPV infection. Vero cells were infected with 5 MOI live CHPV for indicated time or with heat-inactivated (56 °C/20 min) CHPV (HI-CHPV) for 7 h. Cells were observed under a bight field microscope for cytopathic changes and images were collected. The same cells were further immunostained with anti-CHPV-N antibody. Scale bar = 20 µm. **Figure S1B:** Graph showing percentage of cells in Figure S1B with round morphology and percentage of cell death evaluated by MTT assay, with mean ± SD from three independent experiments. **Figure S1C:** Western blot detection of the ectopic expression of FLAG-tagged CHPV-N protein using a rabbit polyclonal anti-FLAG antibody. **Figure S1D:** Graph showing correlation between CHPV-N and CHPV-L puncta in immunostaining (Figure 1B) by plotting Pearson’s correlation coefficient for each time point (n = 3). *p* < 0.5 (*), *p* < 0.001 (***) in Student’s *t*-test. **Figure S2A:** Absence of co-localization between CHPV-IBs and mock plasmid ectopically expressing GFP. Vero cells were transfected with GFP-only vector and, after 24 h, infected with 1 MOI CHPV for 4 h. Cells were then fixed with 4% PFA and immunostained for CHPV-N (blue) and TIA-1 (red), showing GFP does not co-localize with the IBs in contrast to GFP-G3BP1 which co-localizes with CHPV-IBs (Figure 2J). Scale bar = 10 µm. **Figure S2B:** Graph shows the distribution of signal intensities in arbitrary units (A.U.) for TIA-1, GFP and CHPV-N across a line indicated by red arrow, using the ImageJ software. **Figure S2C:** Absence of co-localization between lipid droplets (LD) and CHPV-IBs. Vero cells were infected with 1 MOI CHPV for 4 h. Cells were then fixed with 4% PFA and immunostained for LDs using BODIPY D493 stain and CHPV-N antibody. **Figure S2D:** Graph shows the distribution of signal intensities in arbitrary units (A.U.) for LDs and CHPV-N across a line indicated by red arrow, using the ImageJ software. **Figure S2E:** Uninfected N2A cells stained with TIA-1 (red, upper panel) and eIF3η (red, lower panel) to serve as background control for Figure 2L. The nuclei were counterstained with Hoechst dye. Scale bar = 10 µm. **Figure S2F:** Vero cells were infected by CHPV 1 MOI for 4 h to induce IBs. Cells were fixed with 4% PFA and then processed for IFA to detect CHPV-N/GAPDH for CHPV-IBs. The nuclei were counterstained with Hoechst dye. Scale bar = 10 µm. **Figure S2G:** Graph shows the distribution of signal intensities in arbitrary units (A.U.) for GAPDH and CHPV-N across a line indicated by red arrow, using the ImageJ software. **Figure S2G:** Absence of co-localization between CHPV-IBs and GAPDH. Vero cells were infected with 1 MOI CHPV for 4 h. Cells were then fixed with 4% PFA and immunostained for CHPV-N (green) and GAPDH (red), showing GAPDH does not co-localize with the IBs. Scale bar = 10 µm. **Figure S3A:** The schematic shows outline of experimental design. Vero cells grown on coverslips pretreated with CHX (50 μg/mL) for 1hr. Both untreated and treated cells were incubated with 1mM SA for 40 min in the absence or presence of CHX, respectively. **Figure S3B:** Cycloheximide (CHX) treatment inhibits SA-induced SGs. Vero cells were left untreated or pretreated with CHX (50 μg/mL) for 1h. Both untreated and treated cells were incubated with 1mM SA for 40 min in the absence or presence of CHX, respectively, and were fixed with 4% PFA and immunostained for TIA-1 (red) and PABP1 (green). The nuclei were counterstained with Hoechst dye. Scale bar = 10 µm. **Figure S3C:** Graph shows average number of SGs formed by SA treatment (~50 SGs/cell) versus almost no SGs formed upon CHX pretreatment. (n = 50). **Figure S4A:** Dose optimization of siRNA knockdown of PKR. Vero cells were double transfected with increasing doses of PKR siRNA (25 nM, 50 nM and 100 nM) to optimize efficient knockdown of ~70–80%. **Figure S4B:** Dose optimization of siRNA knockdown of TIA-1. Vero cells were double transfected with increasing doses of TIA-1 siRNA (25 nM, 50 nM and 100 nM) to optimize efficient knockdown of ~70–80%. **Figure S4C,D:** Graphs showing relative intensity of PKR and TIA-1 after knockdown by respective siRNAs and by normalizing with tubulin as loading control and NS (Non -specific)-siRNA as internal control. *p* < 0.5 (*), *p* < 0.001 (***), ns = not significant in Student’s *t*-test. **Figure S4E:** Cellular CHPV-N expression is reduced upon siRNA-mediated knockdown of TIA-1/PKR. Vero cells were double transfected with TIA-1/PKR siRNA, infected with 1 MOI CHPV for 4 h and fixed with 4% PFA for immunostaining. The cells were immunostained for CHPV-N and nuclei were counterstained with Hoechst. Scale bar = 10 µm. **Figure S4F:** Quantification of CHPV-N expression upon siRNA-mediated knockdown of TIA-1/PKR. The graph shows significant decrease in percentage of cells expressing CHPV-N after knockdown of TIA-1/PKR (n = 3). *p* < 0.001 (***) in Student’s *t*-test. **Figure S4G,H:** The phase contrast images showing the inhibition of CHPV-induced cytopathic effect (round cell morphology) in Vero cells after siRNA knockdown of TIA-1 or PKR (lower panel, after infection). Before CHPV infection, cells were completely healthy after double transfection of both siRNAs (upper panel, before infection). **Figure S5A:** Vero cells were immunostained without SA treatment to observe the background staining for TIA-1 (red) and PABP1 (green) to serve as control for Figure 5A. **Figure S5B**: Vero cells were infected by CHPV 1 MOI for 4 h to induce IBs. Cells were fixed with 4% PFA and then processed for IFA to detect CHPV-N/PKR for CHPV-IBs. The nuclei were counterstained with Hoechst dye. Confocal z-stack was used to generate the maximum intensity projection of all three channels with 9 sections (from 1.43 µm to −2.57 µm) of images, showing the PKR localizes with CHPV-N puncta. **Figure S6A:** Kinetics of p38 activation with no change in p-PKR/p-eIF2α. Vero cells were infected with live CHPV (1 MOI) for the indicated time. Infection with heat-inactivated (HI) CHPV was performed for 7 h. The phosphorylated forms of eIF2α (p-eIF2α, Ser51), PKR (p-PKR) and p38 (p-p38) were measured by Western blot analysis using phospho-specific antibodies. Total level of these kinases was determined by a pan-antibody. Tubulin served as a loading control. **Figure S6B:** Graphical representation of the relative amount of p-eIF2α, eIF2α, p-PKR, PKR, p-p38 and p38 protein in each sample after normalizing to tubulin was plotted over the no-virus control in lane 1, set as 1. The error bar indicates mean ± SD (n = 3). *p* < 0.01 (**), *p* < 0.001 (***) in Student’s *t*-test. **Video S1:** A 3-D video rotation of confocal z-stack of Vero cells infected with CHPV 1 MOI for 4 h was reconstructed. The cells were immunostained with CHPV-N- and PKR-specific antibodies. The nucleus was counterstained with Hoechst dye. Confocal z-stack was used to generate the maximum intensity projection of all three channels, showing PKR co-localizes with CHPV-N in x, y and z axis. **Video S2:** Vero cells infected with CHPV 1 MOI for 4 h. The cells were immunostained with CHPV-N- and PKR-specific antibodies. The nucleus was counterstained with Hoechst dye. A video from 9 confocal z-stack images was reconstructed using lasX software along the z axis, showing PKR co-localizes with CHPV-N puncta.

## Abbreviation

Immunofluorescence assay: IFA, T-cell intracellular antigen: TIA-1, Poly(A)-binding protein: PABP, Argonaute 2: Ago2, RasGTPase-activating protein-binding protein 1: G3BP1, eukaryotic initiation factor 3 eta: eIF3η, eukaryotic initiation factor 2 alpha: eIF2α, interferon inducible protein kinase R: PKR, stress granule proteins; SGPs, lipid droplets: LDs, sodium arsenite: SA.
