# Supplementary figures and images for: Chandipura Virus Forms Cytoplasmic Inclusion Bodies through Phase Separation and Proviral Association of Cellular Protein Kinase R and Stress Granule Protein TIA-1
Source: Viruses. 2024 Jun 26;16(7):1027. doi: 10.3390/v16071027 (PMC11281494; doi:10.3390/v16071027)

# Supplementary Figure 1

(A)

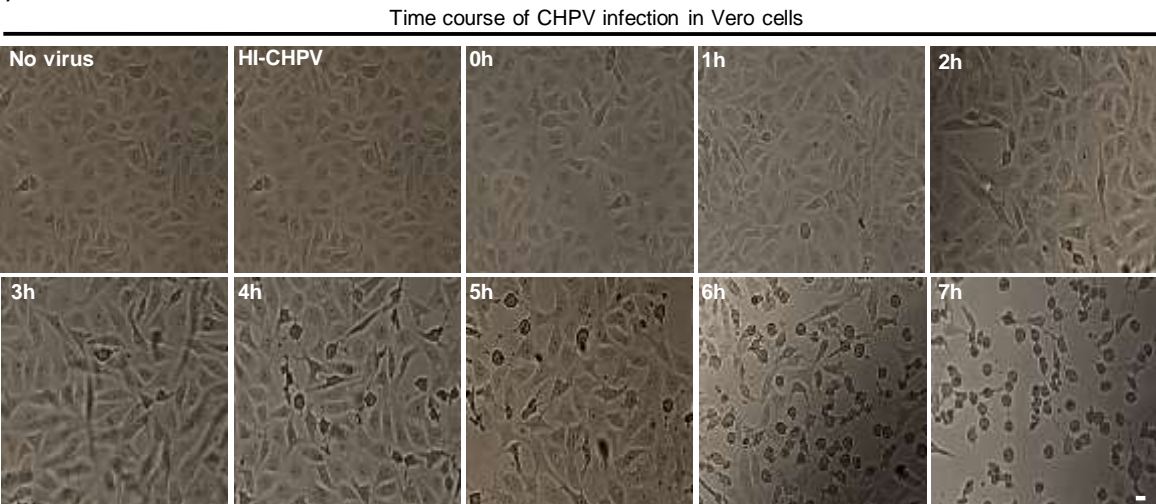

(B)

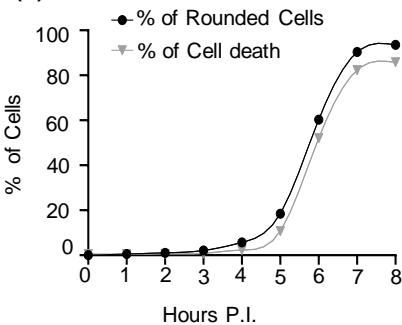

(C)

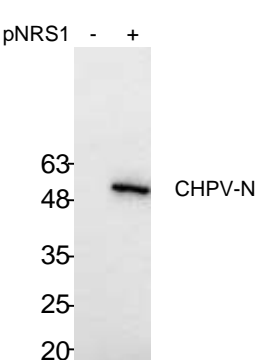

(D)

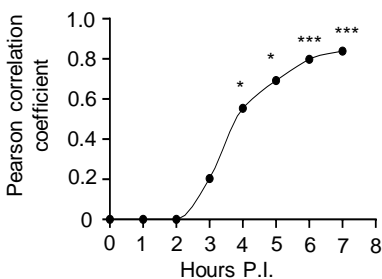

Supplement: Supplementary file 1 [file viruses-16-01027-s001.zip › SUPPLEMENTARY/Figure S1.pdf]

## Supplementary Figure 2

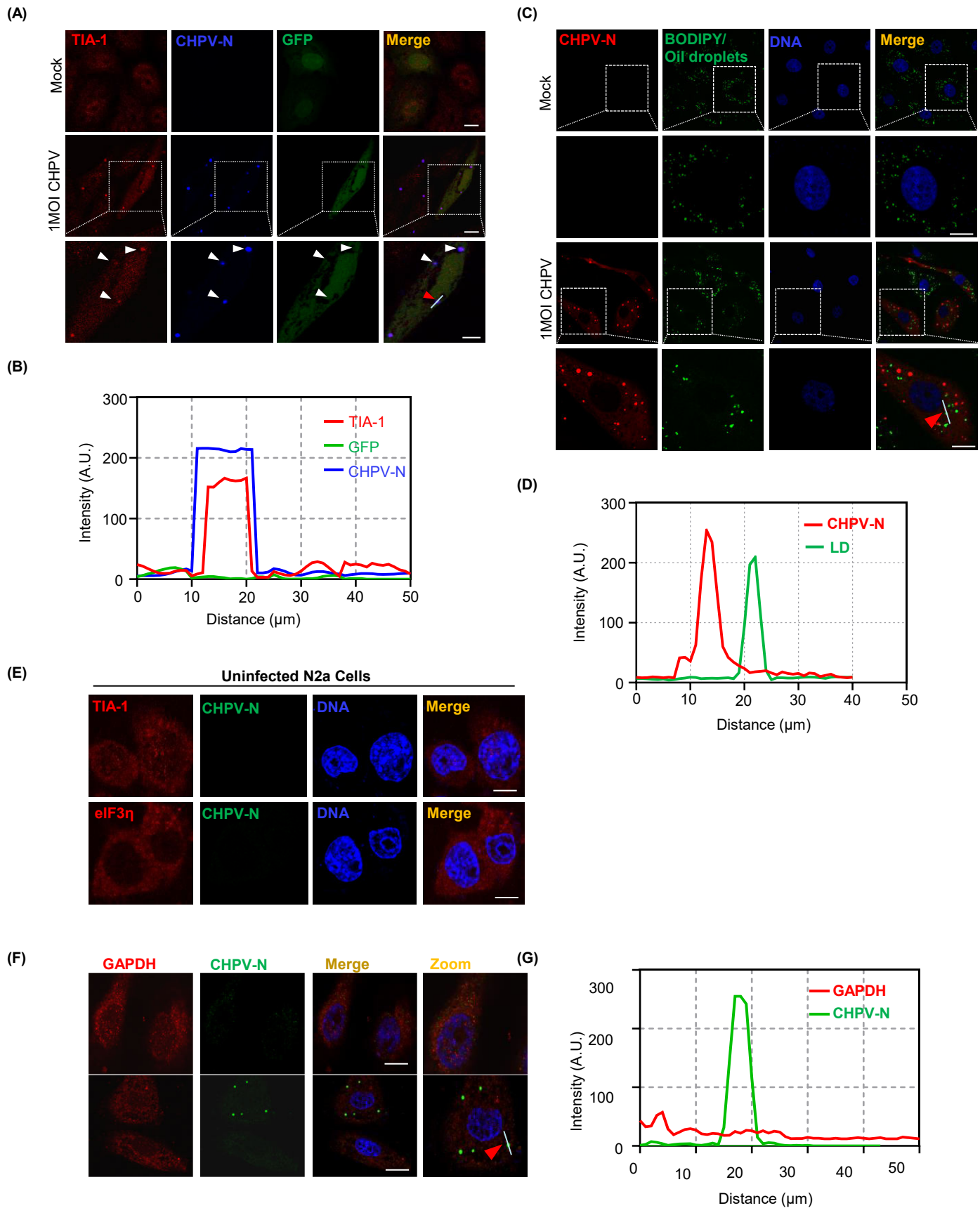

Supplement: Supplementary file 1 [file viruses-16-01027-s001.zip › SUPPLEMENTARY/Figure S2.pdf]

# Supplementary Figure 3

(A)

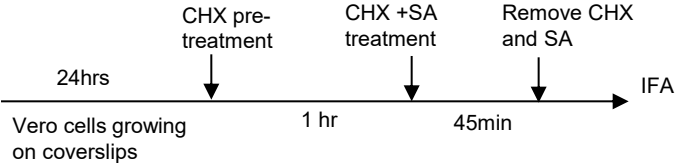

(B)

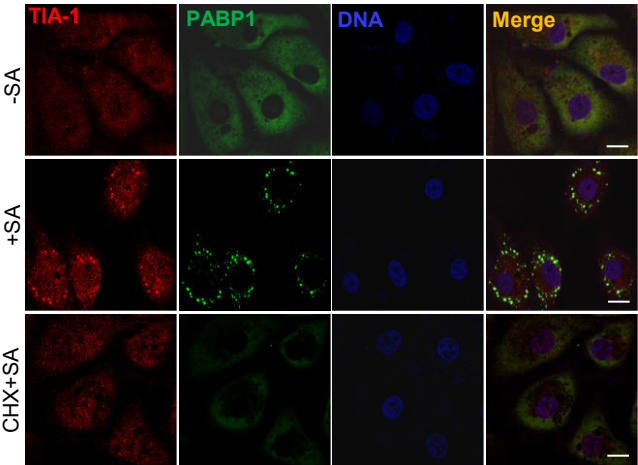

(C)

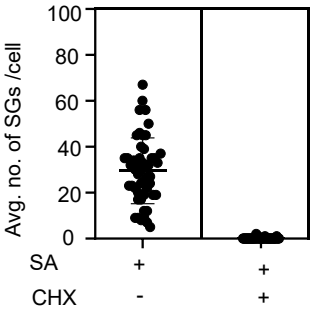

Supplement: Supplementary file 1 [file viruses-16-01027-s001.zip › SUPPLEMENTARY/Figure S3.pdf]

# Supplementary Figure 4

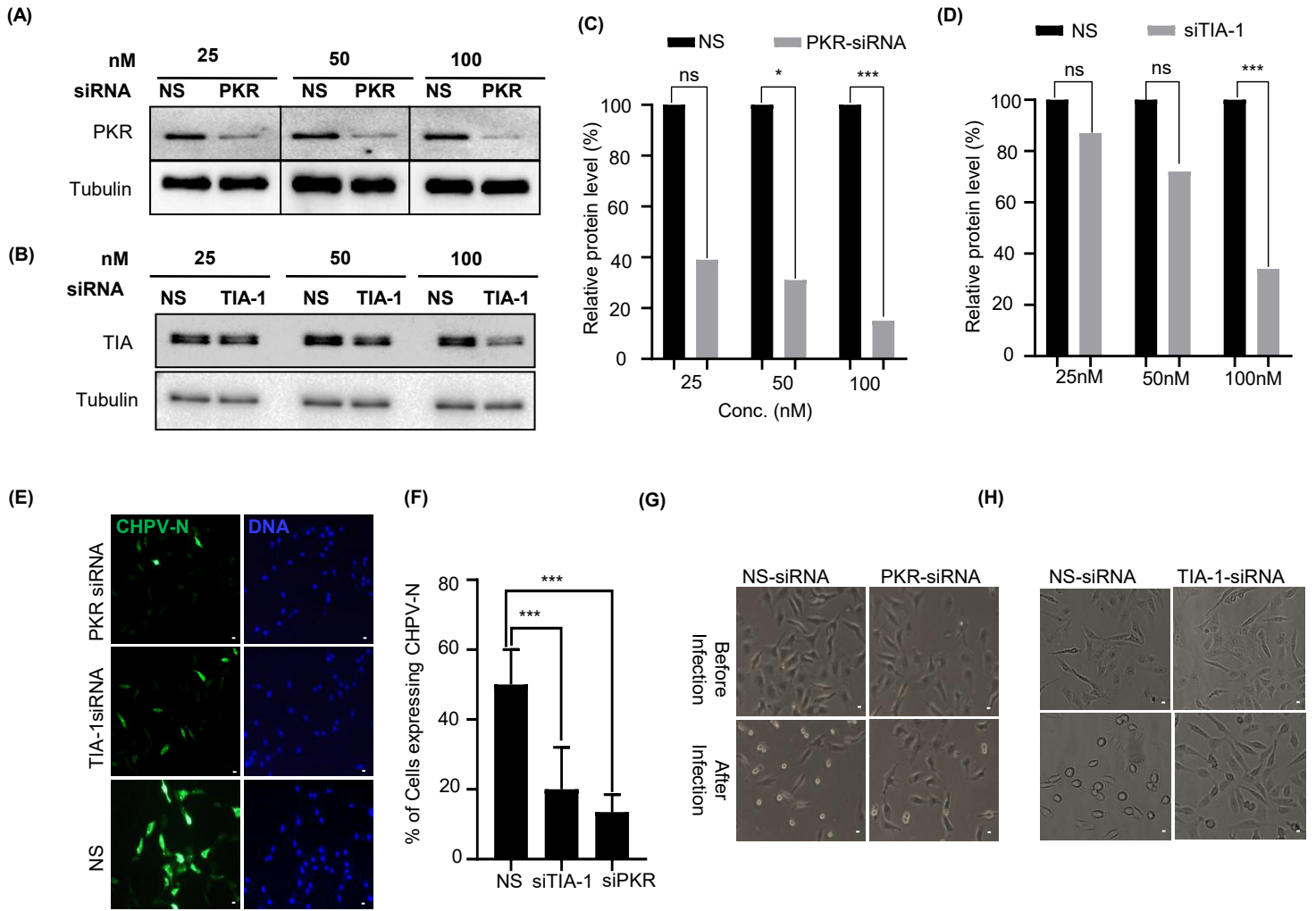

Supplement: Supplementary file 1 [file viruses-16-01027-s001.zip › SUPPLEMENTARY/Figure S4.pdf]

# Supplementary Figure 5

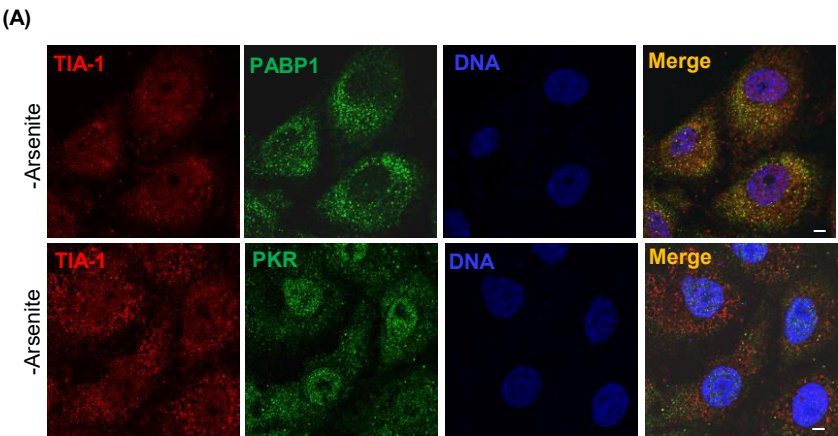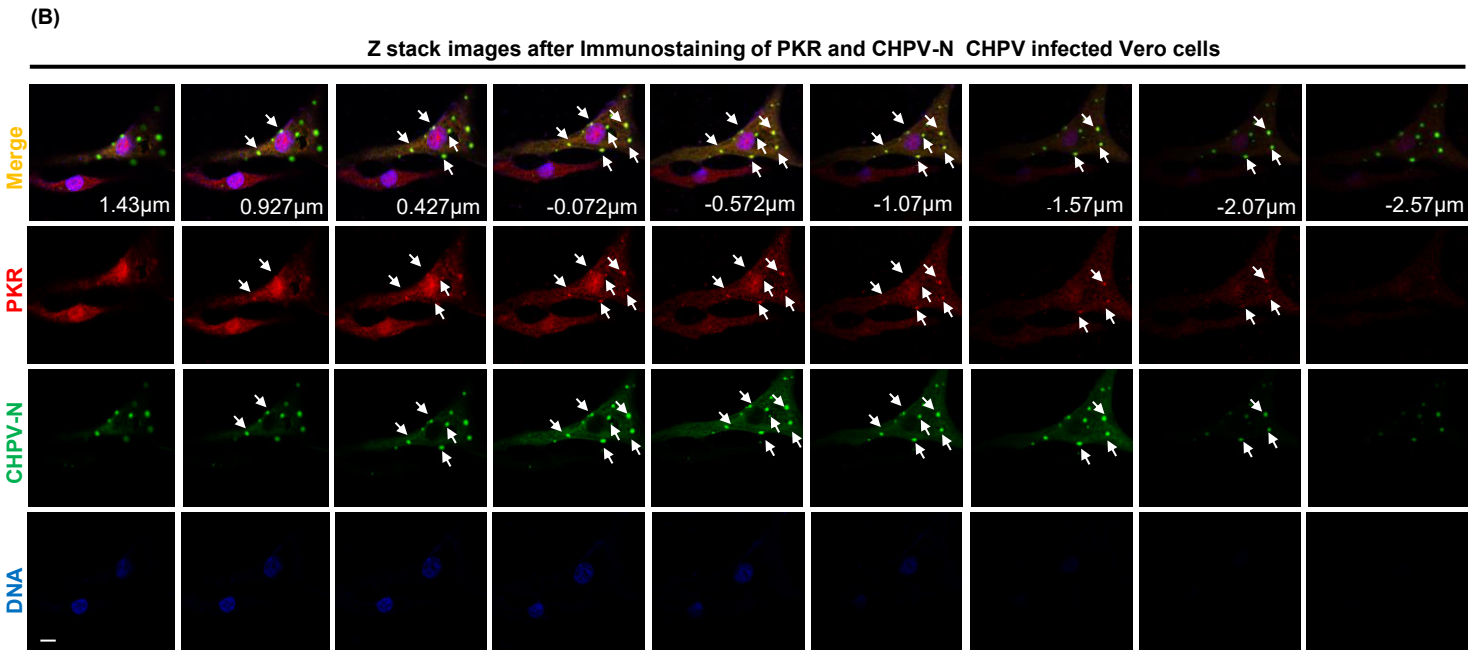

Supplement: Supplementary file 1 [file viruses-16-01027-s001.zip › SUPPLEMENTARY/Figure S5.pdf]

# Supplementary Figure 6

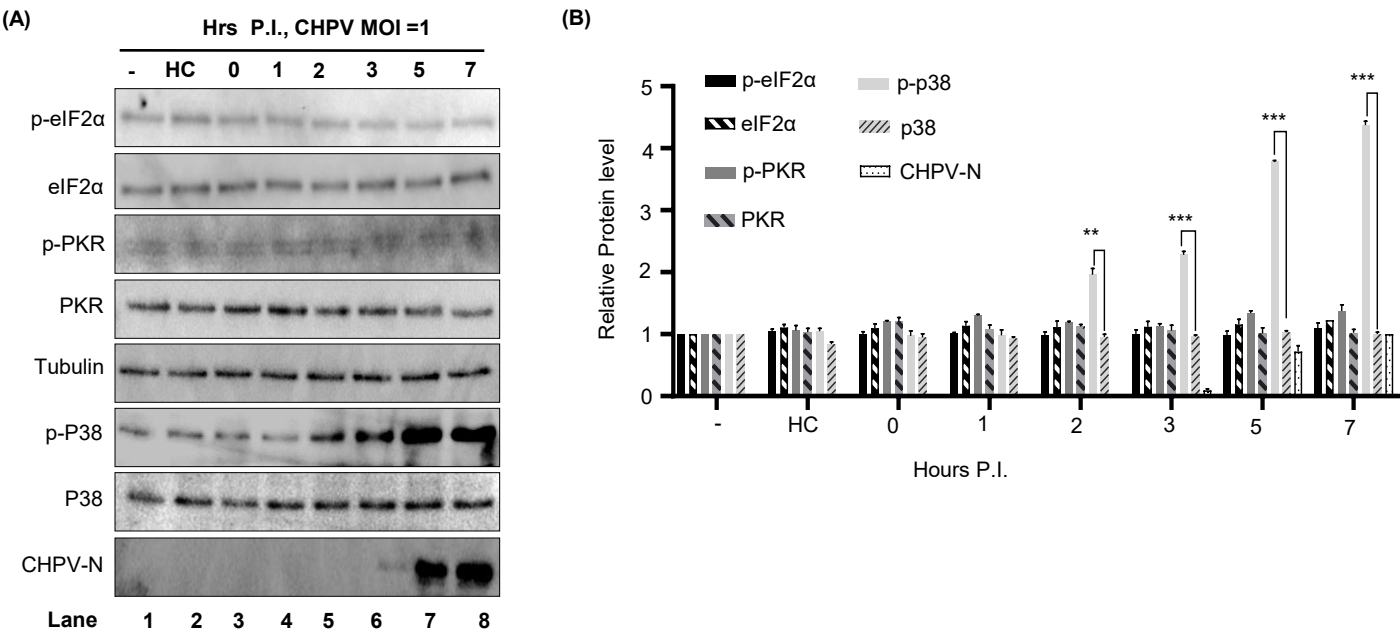

Supplement: Supplementary file 1 [file viruses-16-01027-s001.zip › SUPPLEMENTARY/Figure S6.pdf]
